# Methodological Considerations in Developing a Prognostic Nomogram for Atrophic Scar Outcomes Following Fractional Laser Therapy

**DOI:** 10.1111/jocd.71187

**Published:** 2026-09-06

**Authors:** Ume Aiman, Muhammad Salar, Vishan Das

**Affiliations:** ^1^ Liaquat University of Medical and Health Science Jamshoro Jamshoro Sindh Pakistan; ^2^ Chandka Medical College Larkana Sindh Pakistan

To the Editor,

We read with great interest the recently published article entitled “Risk Factors and Nomogram Model for Poor Prognosis in Atrophic Scar Patients Treated With Fractional Laser Therapy: The Role of Nursing Care in a Retrospective Cohort Study” by Li et al. [1]. The authors are commended for their proposal of a clinically relevant prognostic nomogram that incorporates biological, clinical, and psychosocial factors to aid in the individualisation of the risk level after FL therapy. The use of nursing‐related variables in prognostic modeling represents an interesting multidisciplinary approach and reflects the current trend towards individual care in aesthetic dermatology.

However, there are a number of methodological points that could be explored further. First, although the model has good discriminatory performance (training AUC = 0.96; validation AUC = 0.93), it was trained on a relatively small retrospective cohort that had a large number of candidate predictors. This outstanding performance suggests that the model may be overfitting the data, meaning that the accuracy of predictions made by the model may be too high and may not be reproducible in independent populations, especially if the sample size is too small for the model to be well developed [2, 3]. In addition to discrimination, assessment of model calibration is important to determine how closely the predicted probabilities correspond to the observed outcomes [2, 4]. Second, the authors conducted split‐sample validation, which involved randomly splitting the already limited data set; however, this method is generally not recommended as a validation strategy because it decreases the statistical power of the study, and it provides only limited information about model transportability. Current prediction‐model methodology suggests using bootstrap resampling or repeated cross‐validation to validate the model in the same sample the model was developed from and to test the model in a new external sample before clinical use [2, 4].

In addition, the study does not provide a clear definition for ‘poor prognosis’, which may affect the reproducibility and the risk of misclassification of the outcome [2, 4]. Also, the candidate predictors were chosen based on univariate statistical significance before multivariable analysis, which has been long discouraged as it can lead to overlooking clinically relevant predictors and increase model instability and selection bias [4, 5]. Lastly, while the manuscript complies with the STROBE guideline, the reporting of the prediction‐model development studies should be done in a manner that is compliant with the Transparent Reporting of a Multivariable Prediction Model for Individual Prognosis or Diagnosis (TRIPOD) statement, which recommends the reporting of predictor selection, model specification, calibration, validation, and clinical implementation to facilitate transparency, reproducibility, and clinical interpretability [2, 4].

Larger multicenter cohorts with appropriate sample size would be useful in the future to reduce overfitting and improve representativeness [3]. Internal validation should be performed preferably using the bootstrap resampling method or repeated cross‐validation and extensive external validation in geographically and ethnically different patient cohorts before clinical use [2, 4]. Where possible, outcome measures should be specified in accordance with validated instruments of scar assessment with pre‐specified thresholds and blinded assessment [2, 4]. In addition, predictor selection should be based on biological plausibility and available clinical data and not just univariate significance testing [5]. Following TRIPOD guidelines would further enhance the methodological transparency, independent validation, reporting of calibration, and the confidence levels of the clinical usefulness of prognostic nomograms in aesthetic dermatology [2, 4].

Finally, Li et al. [1] have created an innovative and clinically relevant prognostic model which highlights the need for a multidisciplinary approach to the management of atrophic scars. Consideration of the methodological issues mentioned above will further enhance the reliability, replicability, and transferability of the proposed nomogram, which will help to make it faster and more easily adopted in routine clinical practice. We hope these comments are taken in a positive spirit and will help further improve prediction modeling in dermatologic research.

## Funding

The authors have nothing to report.

## Conflicts of Interest

The authors declare no conflicts of interest.

## Data Availability

Data sharing not applicable to this article as no datasets were generated or analysed during the current study.
